# *Chara zeylanica* J.G.Klein ex Willd. (Charophyceae, Charales, Characeae): First European Record from the Island of Sardinia, Italy

**DOI:** 10.3390/plants10102069

**Published:** 2021-09-30

**Authors:** Ralf Becker, Hendrik Schubert, Petra Nowak

**Affiliations:** 1 Independent Researcher, 26121 Oldenburg, Germany; becker.r@posteo.de; 2 Aquatic Ecology, Institute of Biosciences, University of Rostock, 18059 Rostock, Germany; hendrik.schubert@uni-rostock.de

**Keywords:** charophytes, *Willdenowia*, Sardinia, biogeography, *Chara zeylanica*

## Abstract

The first record of a species belonging to the genus *Chara* L. subgenus *Chara* R.D.Wood section *Grovesia* R.D.Wood subsect. *Willdenowia* R.D.Wood from Europe is presented here, thus challenging the interpretation of its distribution pattern as an intertropical group of charophytes. The morphological characters of the specimens, as well as the results of a phylogenetic analysis, clearly identified them as *Chara zeylanica* J.G.Klein ex Willd. Although the subsection *Willdenowia* has yet to receive a thorough taxonomic treatment, a discussion of its relationship to other taxa of this subsection is provided despite the lack of a commonly agreed upon taxonomic concept. The ecological conditions of the Sardinian site of *C. zeylanica* are presented. Moreover, the status of and threats to this taxon, and hypotheses regarding potential pathways through which it reached Europe, are discussed.

## 1. Introduction

Charophytes are morphologically complex macrophytic green algae with a worldwide distribution. Because they are close relatives of the earliest land plants [1], they have attracted growing scientific interest in recent decades. However, in addition to becoming a subject of academic interest, charophytes play a major role in bioindication systems due to their species-specific pattern of niche occupation [2,3]. Moreover, Characeae are among the most threatened groups of organisms on earth [4,5,6], and have thus been targeted by nature conservation actions [7,8,9]. As charophytes occur in an astonishingly wide variety of habitats, ranging from ultraoligotrophic freshwater to hypersaline and hypertrophic environments, their presence is often measured in water quality assessments and other related fields [10,11].

For the development of such bioindication systems, having comprehensive and reliable knowledge about the habitat preferences and distribution patterns of the individual species is essential, as is the accurate identification of charophyte species, and the formulation of a sound taxonomic concept. In recent decades, a large number of studies have attempted to fulfil these requirements [12,13,14,15,16,17,18,19]. As a result, our knowledge about the biogeography of charophytes has increased substantially. However, whereas in the past site-specific information about the occurrence of the individual species was provided [20], recent treatments have led to the development of large-scale distribution grid maps and detailed descriptions of the species’ preferred habitat conditions [21].

For several species, a strong correspondence between the distribution range and the niche structure was found. For example, the strictly circumpolar distribution of *Tolypella normaniana* Nordst. can be explained by its temperature preference (cold-stenothermic). Moreover, it has been shown that species such as *Chara vulgaris* L. or *C. braunii* C.C.Gmelin occur in a broad range of habitats on all continents, except for Antarctica [22]. 

However, unresolved questions regarding charophytes have hampered the development of general bioindication schemes that are also applicable outside of the reference regions, which have mainly been restricted to specific geographic scales [3]. One of these questions related to the absence of subsection *Willdenowia* R.D.Wood (section *Grovesia* R.D.Wood) in Europe is dealt with here, as we provide the first record of the presence of *Chara zeylanica* J.G.Klein ex Willd. in Europe from the Mediterranean island of Sardinia (Italy). 

In a global taxonomic treatment of charophytes by Wood [23,24], the genus *Chara* L. was divided into five sections with a total of eight subsections. Subsection *Willdenowia* comprises diplostephanous species with triplostichous cortication and a completely ecorticated basal branchlet segment. According to Wood [23,24]), this subsection includes just one species, *Chara zeylanica*, which has several varieties and forms. This approach was not universally accepted because it combined A) monoecious and dioecious taxa, B) monoecious taxa with sejoined and conjoined gametangia, and C) taxa with tetra- and octoscutate antheridia [23]. However, several authors used Wood’s concept as a basis for investigating the distribution pattern of subsect. *Willdenowia,* and came to the conclusion that it can best be described as an intertropical taxon [25,26]. On the other hand, as distinct patterns of the distribution of subspecies and varieties of *Chara zeylanica sensu* Wood [23] emerged, a fine-resolution taxonomic treatment of subsection *Willdenowia* was clearly needed for biogeographical purposes [25,27]. In an approach designed to overcome the problems caused by Wood’s taxonomic concept, van Raam [28,29] presented an alternative view in which subsect. *Willdenowia* was divided into 20 species that were mainly distinguished by the abovementioned criteria of gametangia position, antheridia morphology, and sexuality.

However, irrespective of which concept was applied, neither *Chara zeylanica* nor any other taxon of subsect. *Willdenowia* has previously been recorded anywhere in Europe, even though numerous investigations of charophytes have been performed throughout the Mediterranean area in recent decades [30,31,32,33,34,35,36]. *Chara zeylanica* occurs mainly in tropical and subtropical regions of the world [23,37,38,39,40,41,42,43]. As it is an “intertropical taxon”, the first record of the presence of *C. zeylanica* in Europe could be considered a surprise. On the other hand, Corillion and Guerlesquin [26] and Proctor et al. [25] have reported, taxa of subsect. *Willdenowia* have been found in North America at up to 45° N under climatic conditions comparable to those in Northern Europe. There are historical records of the presence of the species from Egypt and Israel [26,44], as well as reports of extinct occurrences from Algeria [45]. Consequently, limitations other than climatic conditions should be responsible for the failure to observe the presence of taxa of subsect. *Willdenowia* in Europe, which is a well-investigated region that certainly cannot be regarded as undersampled. Recent records of the presence of non-native charophyte species with predominantly tropical and subtropical distributions—such as reports of the presence of *Chara fibrosa* C.Agardh ex Bruzelius ssp. *benthamii* (A.Braun) Zaneveld or *Chara* c.f. *chrysospora* J.Groves and Stevens in rice fields, lakes, and an artificial stormwater retention pond in Southern France, Italy, and Crete, respectively [32,46,47,48]—indicate that the climatic conditions in the Mediterranean area are suitable for the establishment of intertropical taxa.

The main aim of this study is to document the first record of the presence of *Chara zeylanica* in Europe, and the morphological features of the Sardinian specimens we collected. Moreover, this study contributes to knowledge about the taxonomic classification, the ecological requirements, and the geographic distribution of this mainly tropical and subtropical species. To support our morphological analysis, we used *rbcL* and *matK* barcodes, as previous barcoding studies have shown that a combination of these sequences is suitable for investigating species of the genus *Chara* [49,50,51,52].

## 2. Results

### 2.1. Ecology

*Chara zeylanica* can colonize a broad range of both brackish and freshwater habitats throughout the tropical and subtropical zones of the world. These habitats include permanent and temporary bodies of water, such as lakes, ponds, pools, ditches, temporarily flooded wetlands, canals, rice fields, and retention ponds [26,38,40,41,42,43]. Few of the existing hydrochemical datasets cover a spectrum ranging from low-impacted waterbodies with total P-concentrations below 20 µg L^−1^ and total N-concentrations between 0.425 and 1.9 mg L^−1^ [43] to eutrophic habitats [37]. According to Muller et al. [45], *C. zeylanica* needs temperatures of approximately 25 °C for fructification.

**Figure 1:**
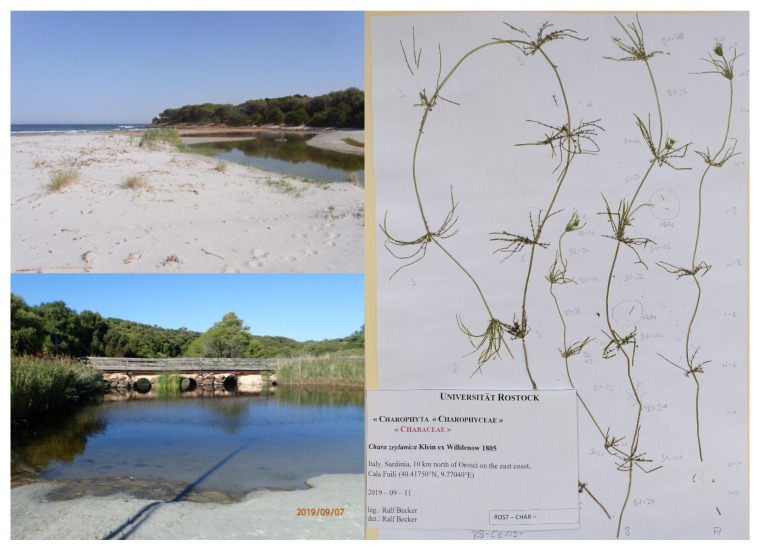
Habitat photographs (**left panels**) and habitus (**right panel**) of specimens collected at Cala Fuili, Sardinia. Plants were collected in the widening of the small stream just in front of the bridge.

The only European site (reported here for the first time) where the presence of *C. zeylanica* has been detected is at Cala Fuili, which is located north of Orosei on the east coast of Sardinia, Italy (coordinates: 40°25′03″ N, 9°46′13″ E; coordinate system WGS 84) (Figure 1). The specimens were found in September 2019 at a depth of about 1 m, mainly in sandy to silty places with stony substrate, in a shallow and probably permanent small stream located close to the beach, or 110 m from the Mediterranean Sea. The specific site where the *C. zeylanica* specimens were found was situated directly next to a bridge (Figure 1, left image below), and was therefore disturbed by the structure. By contrast, the neighbouring stream sections and landscape areas can be considered semi-natural habitats. The small population of *C. zeylanica* was observed to have high fertility, with ripe antheridia, oogonia, and oospores. The nutrient conditions at the sampling date were as follows: NH_4_-N 0.108 mg L^−1^, NO_3_-N 0.279 mg L^−1^, total N 1.143 mg L^−1^, PO_4_-P 0.073 mg L^- 1^, and total P 0.137 mg L^−1^. The water hardness was 26.4 °dH (Ca 62.2 mg L^−1^, Mg 76.8 mg L^- 1^), pH 8.3. Although the salinity at the sampling date was 1.9, the salinity of the site probably varies because it is close to the coast. At the same site in May 2016, a salinity level of 4.4 was recorded and the Cl concentration was found to be 2819 mg L^−1^, instead of 1290 mg L^- 1^, as measured in September 2019.

### 2.2. Morphological Description

**Figure 2:**
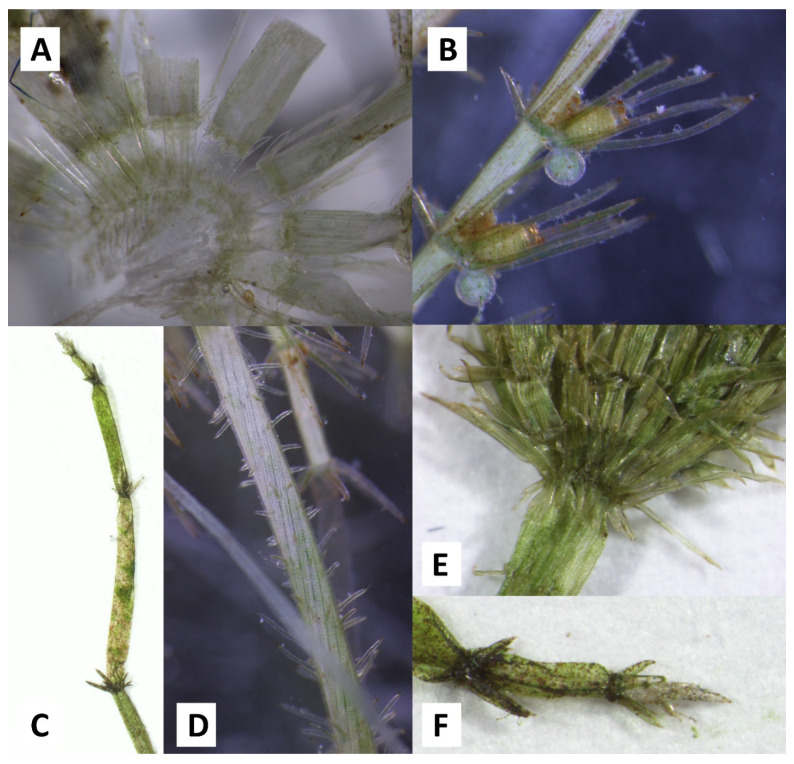
Detailed photographs of *C. zeylanica* collected at Cala Fuili, Sardinia. (**A**) branchlet whorl with ecorticated basal segments; (**B**) conjoined gametangia; (**C**) ecorticated end segments with bract cells; (**D**) triplostichous main axis cortication with single spines; (**E**) diplostephanous stipulodes; (**F**) branchlet tip cell, surrounded by bract cells.

The specimens are 30–60 cm long, erect and straight, stout, fresh to greyish green, and slightly incrusted (Figure 2). The main axis diameter is 589–1076 µm with a mean value of 844 µm, slightly (0–4) branched. Most of the internodes are 4.4–8.0 cm long, and are usually much longer (up to 4 ×) than the branchlets. The uppermost 1–2 internodes are only 0.5–2.2 cm long, and are generally shorter than the adjacent branchlets. The cortex is usually triplostichous, and is rarely (partly) diplostichous and tylacanthous to isostichous (Figure 2D). Single, acute, thin, and needle-like spines are observed on the young internodes, and rarely on the older internodes. These spines can vary in length (182–1468 µm long) even on the same plant, and mainly point downwards (Figure 2D). The stipulodes are acute, elongated, and well developed. They are arranged in two regular tiers with two pairs per branchlet (Figure 2E). The upper stipulodes are longer than the lower ones. As the upper stipulodes are 515–1045 µm long (a mean value of 760 µm), they are usually longer than the diameter of the axes, and are much longer than the lowermost branchlet segment. The lower stipulodes are sometimes of unequal lengths, at 161–475 µm long, with a mean value of 293 µm. The branchlets are 9–12 in whorl and generally much shorter than the internodes, at 2.0–4.3 cm long. The branchlets of the uppermost 1–2 youngest whorls are even shorter, at just 0.2–2.0 cm long. The lowermost basal segments of the branchlets are ecorticated, and are very short at 208–479 µm long (mean value 343 µm) and 189–470 µm wide (mean value 318 µm). These segments are hidden behind the upper stipulodes (Figure 2A). The branchlets consist of 7–10 segments, with the lowermost segments always being ecorticated, followed by 4–6 corticated segments and 2–5 ecorticated distal segments with a tiny acute end cell on top, surrounded by a ring of bract cells (Figure 2C,F). The bract cells (5–8) are well developed (220–843 µm long), slender, and acute, and are shorter than the bracteoles. The two bracteoles are very long (990–1948 µm), at 1–2.5 × longer than the oogonia and oospores (Figure 2B). All of the fertile specimens are monoecious with conjoined gametangia (Figure 2B). Gametangia usually occur only at the nodes of corticated segments, and are rarely observed at the lowest nodes just above the ecorticated segment. The gametangia are mainly solitary, and very rarely geminate. The oogonia are elliptical to elongated oval in shape, are yellow or greenish in colour, and generally have constricted coronulae. The length of the oogonia (without coronula) is (600) 700–850 (900) µm, and the width of the oogonia is 417–575 (600) µm. The length of the coronula is 69–125 (150) µm, and the width of the coronula is (127) 160–200 (250) µm. The oospores are elliptical in shape and black in colour, with a length of (539) 600–685 µm, a width of 375–475 (500) µm, and 10–13 striae. The antheridiae are tetrascutate with a diameter of (300) 350–400 (450) µm. The dried specimens are stored at the herbarium of the University of Rostock (ROST).

### 2.3. Phylogenetic Analysis

The three individuals collected on Sardinia had identical *rbcL* and *matK* sequences. The BLAST of the GenBank nucleotide collection under default settings with *rbcL* from the Sardinian samples as query sequences matched the individuals to *C. zeylanica* from New Caledonia (AB440257) with 100% identity. One basepair (bp) substitution (99% identity) was detected for two further *C. zeylanica* (HQ380481: Sri Lanka, AY720934: Taiwan), but also for a sequence belonging to *C. hydropitys* Rchb. (HQ380464: Puerto Rico).

The BLAST of the GenBank nucleotide collection using *matK* from the Sardinian samples as query sequences matched the individuals with 99% identity (1 bp substitution) to *C. zeylanica* from Myanmar (MT739758). *Chara guairensis* R.M.T.Bicudo (KY656924) and *C. hydropitys* (KY656921) differed from the Sardinian samples by 15 bp substitutions (98% identity), respectively.

**Figure 3:**
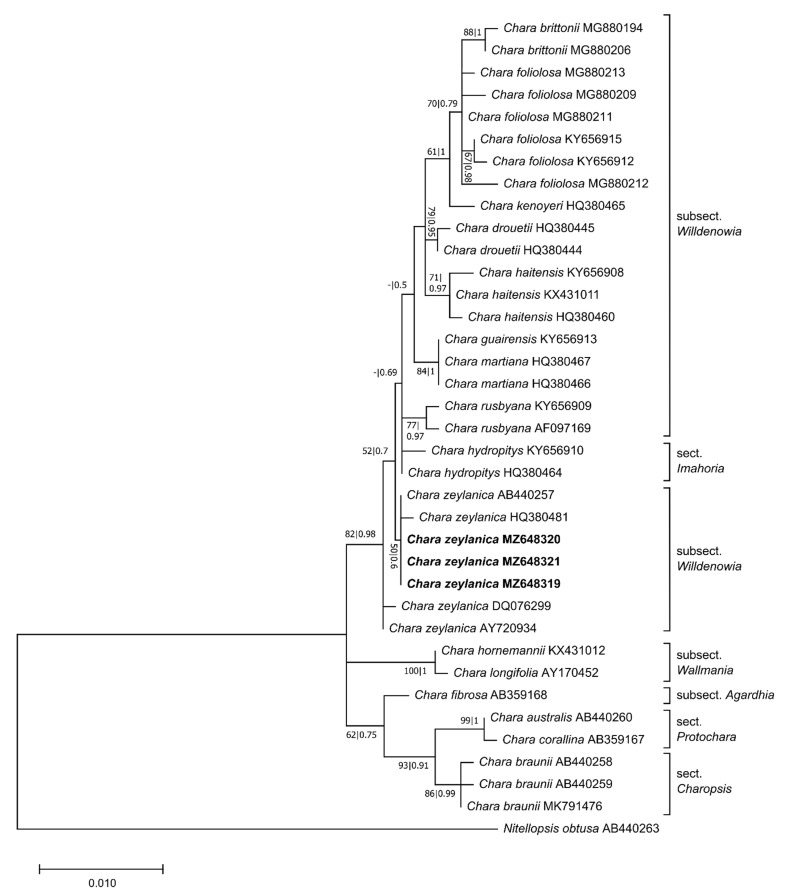
Phylogenetic tree based on 1051 base pairs (bp) of the *rbcL* sequences. The evolutionary history was inferred using the GTR+G + I model. Maximum likelihood bootstrap values (>50%, ML, **left**) and Bayesian posterior probabilities (>0.5, BI, **right**) are given at the branches. The scale indicates sequence divergence in percent. The specimens collected in Sardinia are marked bold. Information about the section and the subsection of the species is given at the tree [24,29,53].

Phylogenetic analyses were performed for *rbcL* and *matK* separately to confirm the species identified through the BLAST search. The final *rbcL* alignment was trimmed to 1051 bp. Within the subsect. *Willdenowia*, 30 variable sites were identified. In the *rbcL* tree (Figure 3), the relationships within the subsect. *Willdenowia* were ambiguous, because several nodes did not have significant supports. The specimens from Sardinia formed a cluster together with *C. zeylanica*, but only with a low level of support (BS: 50%, PP: 0.6). The phylogeny based on the *rbcL* gene sequences only could not be resolved, and relationships of *C. zeylanica* to other species of subsect. *Willdenowia* were ambiguous.

The final *matK* alignment was trimmed to 970 bp. Within the subsect. *Willdenowia*, 64 variable sites were identified. Phylogenetic analysis of the *matK* alignment provided strong bootstrap support for the sequences from the Sardinian samples forming a monophyletic clade with *C. zeylanica* sequence: MT739758 (BS: 100% and BP: 1, Figure 4). The *matK* phylogeny assigned the Sardinian specimens to *C. zeylanica*, and differentiated them from other species of subsect. *Willdenowia* (*C. guarensis*, *C. rusbyana* M.Howe, *C. haitensis* Turpin, *C. foliolosa* Muhl. ex Willd.) and sect. *Imahoria* (*C. hydropitys*). 

## 3. Discussion

### 3.1. Taxonomical Remarks

The specimens collected at Cala Fuili (Sardinia) were shown to qualify, based on their morphological characters, as a taxon belonging to subsect. *Willdenowia* because they are diplostephaneous with triplostichous cortication and have ecorticated basal segments of otherwise corticated branchlets [23]. Following the approach of van Raam [28], who distinguished 20 species within subsect. *Willdenowia*—in contrast to Wood [23], who identified a monospecific subsection—the question of to which species the specimens belong is discussed in detail below.

Van Raam [28] analyzed systematically the problem of gymnopodial (ecorticated first branchlet segment) taxa of the genus *Chara* L. using a stepwise approach. A total of 37 taxa of the genus *Chara* were found to share the character of an ecorticated basal branchlet segment. We recall that a taxon is a taxonomic unit of any rank, which can be species, but also varieties and forms. Eight taxa from subsect. *Willdenowia* can be excluded because they are haplostephaneous (and can thus be assigned to sect. *Imahoria* J. van Raam). Of the remaining 29 diplostephaneous taxa, *C. kenoyeri* M.Howe and *C. rusbyana* can be excluded here because they are dioecious. As a haplostichous species, *Chara pseudohydropitys* Imahori belongs to section *Aghardia* R.D.Wood, and can also be excluded here. Similarly, *C. foliolosa*, *C. tenuifolia* (Allen ex R.D.Wood) R.D.Wood, *C. guairensis*, *C. haitensis*, *C. indica* Bertero ex Spreng., *C. martiana* Wallman, and *C. paucicorticata* Cáceres can be excluded because they have octoscutate antheridia. Unlike the specimens described here, *Chara drouetii* (R.D.Wood) R.D.Wood, *C. michauxii* (A.Braun) Kütz., and *C. formosa* C.B.Rob. are characterised by a sejoined gametangia arrangement. *Chara cubensis* Allen, *C. depauperata* Allen, *C. oerstediana* A.Braun, and *C. diaphana* (Meyen) R.D.Wood have fewer than four corticated branchlet segments, whereas all the specimens found in Sardinia have at least four corticated segments. According to van Raam [28], the remaining 12 taxa belong to *C. zeylanica* as varieties or forms based on quantitative characters, such as spine length relative to axis diameter and the length of the stipulodes.

At this stage, we can conclude that the Sardinian specimens fit the character combination of *C. zeylanica*. Because van Raam [28] failed to provide an adequate description of infraspecific taxa beyond offering a series of tables, the specimens discussed here will not be related to varieties or forms. In any case, the specimens clearly do not belong to *C. foliolosa*, which can be found in the northernmost distribution range of subsect. *Willdenowia* in North America [25].

However, a sound comparison between our specimens and specimens described by other authors [39,40,43,54] is still impossible because of the different taxonomic concepts applied. Taking Wood [23] as a basis, many authors [27,45,55] did not take antheridia morphology into account. Thus, it is extremely difficult to compare their data with the recent concept proposed by van Raam [28].

To obtain independent proof of the morphology-based determination, phylogenetic analyses were performed with the regularly used barcode markers *rbcL* and *matK*. The analyses classified independently the individuals from Sardinia along with other *C. zeylanica*. Morphologically similar species such as *C. foliolosa*, *C. haitensis,* and *C. rusbyana* (previously considered to be varieties or forms of *C. zeylanica sensu* Wood) could be excluded through alignment with GenBank sequences. The phylogenetic analyses showed that *C. hydropitys*, a haplostephaneous species belonging to sect. *Imahoria*, is closely related to the abovementioned *Willdenowia* species, consistent with the findings of previous studies [43,56,57]. The phylogenetic relationships between *C. zeylanica* and *C. hydropitis* were not evident based on *rbcL* sequence data. However, the Sardinian samples were shown to have *rbcL* gene sequences identical to those of a *C. zeylanica* individual from GenBank (HQ380481), which made the categorisation unambiguous. The assignment of the specimens to this taxon was supported by the results of a *matK* analysis, which showed that *C. zeylanica* obtained from GenBank (MT739758) formed a monophyletic clade together with the Sardinian specimens [43,56,57]. Thus, the genetic classification based on the *rbcL* and *matK* sequences clearly supported the morphological determination of the individuals collected at Cala Fuili. The phylogeny of the subsect. *Willdenowia* was not the main focus of this study. Nevertheless, in order to test the phylogeny of *Willdenowia* species in future studies, the taxonomic and geographical basis for an analysis should be broadened, and additional molecular data should be gathered.

### 3.2. Status and Threats

Many charophyte species and their habitats are threatened throughout Europe, and are mentioned in several national Red Lists [4]. Sardinia has a key role to play in the conservation of Characeae in the Mediterranean region [30,58,59]. Becker [30] identified numerous Sardinian hotspots for the conservation of charophytes, and proposed specific action plans that mainly focused on Characeae in brackish habitats. The Sardinian site where *C. zeylanica* has been found is in the hotspot area between Orosei and Capo Comino.

In contrast to rare and threatened taxa, introduced non-native species can become invasive and cause ecological damage, as the example of *Nitellopsis obtusa* (Desv.) J.Groves in North America shows [60]. However, the examples of two alien charophyte species with mainly intertropical distribution that were previously introduced into Europe have so far not been found to have any serious environmental impacts. Both species, *Chara fibrosa* (including ssp. *benthamii*) and *Chara* c.f. *chrysospora*, were probably introduced by humans into rice fields in Southern France and Northern Italy through the importation of contaminated rice seeds [32,46,47,61]. Moreover, while the presence of a population of *Chara fibrosa* ssp. *benthamii* was recorded on the Greek island of Crete [48], it appears that it has been extinct since 2010 [62].

*Chara zeylanica* cannot currently be considered an invasive species among the European charophyte flora. For the moment, the Sardinian population is very small, and is limited to a single and relatively isolated location. Although the species has a high rate of fertility in Sardinia, strong dispersal cannot be expected at this stage. Nevertheless, the development of the Sardinian population of *C. zeylanica* should be monitored.

Although the abovementioned intertropical species *Chara fibrosa* and *C.* c.f. *chrysospora* were probably introduced into Europe by anthropogenic factors [47], this is unlikely to be the case for *C. zeylanica*. The Sardinian site is situated more than 100 km away from the nearest rice fields. The surrounding land is used primarily for grazing sheep and small-scale tourism. Other anthropogenic dispersal pathways (e.g., fishery, bathing, or diving) also appear to be unlikely. On the other hand, Sardinia is an important interim stop for birds migrating between Europe and Africa. As the nearest previous records of the presence of *C. zeylanica* are from a Saharan pond in Algeria at least 88 years ago [45 and literature therein], and from Senegal and Egypt [26,27], we assume that the species was introduced into Sardinia by migrating water birds. However, against the backdrop of climate change, future investigations of *C. zeylanica* and other Characeae should consider whether rice fields in Sardinia and throughout the Mediterranean area play a role in the dispersal of the species.

## 4. Material and Methods

### 4.1. Hydrochemical and Morphological Analyses

Hydrochemical analyses were conducted in a laboratory according to standard methods and national DIN norms, as published by Wasserchemische Gesellschaft [63,64,65]. The nutrient concentrations (NH_4_-N, NO_3_-N, total N, PO_4_-P and total P) were measured using a photometer (CADAS 200 by Dr Lange). The cation concentrations (Ca, Mg) were determined by means of an atomic absorption spectrometer (SpectrAA 55 by Varian). The pH values were analyzed using WTW Multi 3510 IDS. The conductivity, salinity, and chloride levels were determined using WTW Cond 3130, with the specific probe being applied in each case.

The morphological analysis was done by means of a stereo microscope (SZX16; Olympus, Tokyo, Japan) equipped with a digital camera for recording photographs.

### 4.2. DNA Barcoding

The total genomic DNA was extracted using the DNeasy Plant Mini Kit (Qiagen, Hilden, Germany) following the manufacturer’s protocol. Partial sequences of the *rbcL* and *matK* genes were amplified using the primers rbcL-1a (5′-TCG TGT AAC TCC ACA ACC TG-3′) and rbcL-1b (5′-TAC TCG GTT AGC TAC AGC TC-3′), and matK-F2 (5′-GAA TGA GCT TAA ACA AGG ATT C-3′) and matK-R1b (5′-GCA GCC TTA TGA ATT GGA TAG C-3′). The PCR tests were performed in a 30 µL reaction volume with a Taq PCR Master Mix (Qiagen, Hilden, Germany) consisting of 2.5 mM MgCl_2_ (final concentration), and 0.5 pmol of each primer. The PCR products were extracted from agarose gels following the protocol of the Biometra-innuPrep Gel Extraction Kit (Analytik Jena, Jena, Germany), and were sequenced directly using a 3130×L Genetic Analyser (Applied Biosystems, New York, NY, USA) with sequencing primers identical to the primers that were used for the PCR reaction. The quality of the chromatograms of the generated sequences were checked using the BIOEDIT software [66]. The nucleotide sequences identified in this study have been deposited in the GenBank (MZ648319- MZ648324).

Sequences from three specimens collected in Sardinia were submitted to the National Center for Biotechnology Information’s (NCBI) Basic Local Alignment Search Tool (BLAST) [67] to allow them to be checked against the nucleotide collection in the GenBank in order to identify other *Chara* sequences with high scoring similarity pairs (HSP) in the NCBI web server. The phylogenetic analysis was performed with the sequence data from the *Chara* specimens collected in Sardinia, and with data on closely related taxa in the GenBank’s nucleotide database (https://www.ncbi.nlm.nih.gov/nuccore) for both the *rbcL* and *matK* sequences separately, because the sequences available in the GenBank were completely different. Alignments were created and trimmed using BIOEDIT software [66]. Identical sequences were merged into one entry. Sequences differing only in length were also reduced to one genotype. If different taxa had identical sequences, they were retained in the alignment (Table 1). The *rbcL* dataset contained 36 sequences belonging to nine species of the subsect. *Willdenowia*, and seven of haplostephanous species belonging to the sect. *Charopsis, Protochara* and *Imahoria*, and to the subsect. *Wallmania* and *Agardhia*. In addition, *Nitellopsis obtusa* was used as the outgroup (Table 1). For the *matK* dataset, the three Sardinien samples of *C. zeylanica* were analysed together with 13 sequences belonging to six species of the subsect. *Willdenowia* and *Agardhia*, and one species of the sect. *Charopsis* and *Imahoria*, respectivly. *Nitellopsis obtusa* was used as the outgroup (Table 1). Phylogenetic trees were created using the Maximum likelihood (ML) method and Bayesian inference (BI) analysis. The best-fit model of sequence evolution was determined using MEGA v. X [68]. The ML method was applied using MEGA v. X [68], with the HKY+G+I model used as the nucleotide substitution model for the *rbcL* dataset, and the GTR+G+I model used for the *matK* dataset. Branch supports were evaluated using 1000 bootstrap replicates (BS). MrBayes 3.2.7 [69] was used for the BI method. Two independent runs with four chains were run for 10 million generations using the MCMC method. Calculations of the consensus tree, including clade posterior probability (PP), were performed based on the trees sampled after the chains converged using Tracer 1.7 [70]. The first 25% were discarded as burn-in.

## Figures and Tables

**Figure 4:**
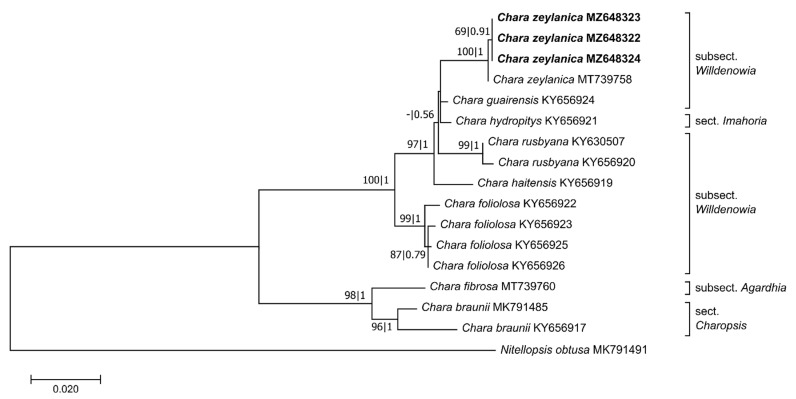
Phylogenetic tree based on 970 base pairs (bp) of the *matK* sequences. The evolutionary history was inferred using the GTR+G+I model. Maximum likelihood bootstrap values (>50%, ML, **left**) and Bayesian posterior probabilities (>0.5, BI, **right**) are given at the branches. The scale indicates sequence divergence in percent. The specimens collected in Sardinia are marked bold. Information about the section and the subsection of the species is given at the tree [24,29,53].

**Table 1:** Sample list of specimens used for phylogenetic analyses. Indicated are the accession numbers of the haplotypes (=non-redundant genotypes) downloaded from the GenBank. Information about the section and the subsection of the species is given in the third column [24,29,53] and identical sequences are given in the last column. n|a = not applicable.

Marker	Species	Section/Subsection	Strain Designation and/or Collection Information	Accession	Reference	Redundant Accessions
*rbc* *L*	*Chara australis*	*Protochara*/-	S002/Unknown	AB440260	[71]	-
	*Chara braunii*	*Charopsis*/-	S036/Japan, Lake Ashino	AB440259	[71]	-
			S019/Japan, Lake Haryu-numa	AB440258	[71]	KJ395929
			GR10-UW37/Greece, Etoloakarnania	MK791476	[59]	-
	*Chara brittonii*	*Grovesia*/*Willdenowia*	KGK2610/USA, Wisconsin	MG880194	[72]	MG880191, MG880186
			KGK3120/USA, New Jersey	MG880206	[72]	MG880205, MG880204, MG880203, MG880202, MG880201, MG880200, MG880199, MG880198, MG880197, MG880196, MG880195, MG880193, MG880192, MG880190, MG880189, MG880188, MG880187, MG880185, MG880184, MG880183
	*Chara corallina*	*Protochara*/-	SK026/Japan, Hiroshima	AB359167	[73]	-
	*Chara drouetii*	*Grovesia*/*Willdenowia*	Proctor Loc 36/Guatemala, San Luis	HQ380445	[74]	-
			KGK0467/Mexico, Quintana Roo	HQ380444	[74]	-
	*Chara fibrosa*	*Agardhia*/*Agardhia*	SK066/ Japan, Hiroshima	AB359168	[73]	AB440261
	*Chara foliolosa*	*Grovesia*/*Willdenowia*	Proctor 138/Mexico	MG880213	[72]	HQ380448
			NY 02146579/USA, Lake Erickson	MG880212	[72]	MG880210, HQ380452, HQ380449
			NY 02145914/USA, Clopper Lake	MG880211	[72]	MG880208, MG880207, KY656911, HQ380451, HQ380447, HQ380446
			NY 00739274/USA, Near New Deal	MG880209	[72]	HQ380450
			SJRP31534/Brazil, Neves Paulista	KY656915	[57]	KY656914, KY656907
			SJRP31929/Brazil, São Paulo	KY656912	[57]	-
	*Chara guairensis*	*Grovesia*/*Willdenowia*	SJRP31523/Brazil, São Paulo	KY656913	[57]	-
	*Chara haitensis*	*Grovesia*/*Willdenowia*	SJRP28306/Brazil, Mato Grosso	KY656908	[57]	-
			NY02145934/Michigan, USA	KX431011	[75]	-
			X-930/USA, Everglades	HQ380460	[74]	HQ380459, HQ380458, HQ380457, HQ380456, HQ380455, HQ380454, HQ380453
	*Chara hornemannii*	*Agardhia*/*Wallmania*	NY00739162/Peru, Lima	KX431012	[76]	-
	*Chara hydropitys*	*Imahoria*/-	SJRP28308/Brazil, Mato Grosso do Sul	KY656910	[57]	-
			KGK0774/Puerto Rico, Lago Carite	HQ380464	[74]	HQ380463, HQ380462, HQ380461
	*Chara kenoyeri*	*Grovesia*/*Willdenowia*	TP118/Panama, Gatun Lake	HQ380465	[74]	-
	*Chara longifolia*	*Agardhia*/*Wallmania*	MB/Canada, Saskatchewan	AY170452	[77]	-
	*Chara martiana*	*Grovesia*/*Willdenowia*	Proctor X-952/Venezuela, Caracas	HQ380467	[74]	-
			Proctor TP097/Brazil, São Paulo	HQ380466	[74]	-
	*Chara rusbyana*	*Grovesia*/*Willdenowia*	SJRP28307/Brazil, Mato Grosso do Sul	KY656909	[57]	KY630506
			LG/ Unknown	AF097169	[75]	AF097168
	*Chara zeylanica*	*Grovesia*/*Willdenowia*	n|a/Taiwan, Gueishan Island	AY720934	unpubl.	-
			n|a/Australia: Elizabeth Creek	DQ076299	unpubl.	-
			S111/New Caledonia	AB440257	[71]	KT343914, KT343913, AB359169, HQ380480, HQ380479, HQ380477, HQ380475, HQ380474, HQ380473, HQ380472, HQ380471, HQ380469, HQ380468
			Proctor X-574/Sri Lanka, Ceylon	HQ380481	[74]	HQ380478, HQ380476, HQ380470
			RB-CZ119A	MZ648319	this study	-
			RB-CZ119B	MZ648320	this study	-
			RB-CT1119-B	MZ648321	this study	-
	*Nitellopsis obtusa*		S042/Germany, Lake Nehmitz	AB440263		MK791482
*matK*	*Chara braunii*	*Charopsis*/-	GR10-UW37/ Greece, Etoloakarnania	MK791485	[59]	-
			48/ Brazil, São Paulo	KY656917	[57]	-
	*Chara fibrosa*	*Agardhia*/*Agardhia*	MY-33/Myanmar, Yezin	MT739760	[43]	MT739765, MT739766, MT739768
	*Chara foliolosa*	*Grovesia*/*Willdenowia*	SJRP31527/Brazil, Paulicéia	KY656925	[57]	-
			SJRP31929/Brazil, São Paulo	KY656923	[57]	-
			SJRP31534/Brazil, Neves Paulista	KY656926	[57]	-
			SJRP28309/Brazil, Mato Grosso do Sul	KY656922	[57]	-
	*Chara guairensis*	*Grovesia*/*Willdenowia*	SJRP31523/Brazil, São Paulo	KY656924	[57]	-
	*Chara haitensis*	*Grovesia*/*Willdenowia*	SJRP28306/Brazil, Mato Grosso	KY656919	[57]	-
	*Chara hydropitys*	*Imahoria*/-	SJRP28308/Brazil, Mato Grosso do Sul	KY656921	[57]	-
	*Chara rusbyana*	*Grovesia*/*Willdenowia*	n|a/Brazil. Mato Grosso do Sul	KY630507	[57]	KY656916
			SJRP28307/Brazil. Mato Grosso do Sul	KY656920	[57]	-
	*Chara zeylanica*	*Grovesia*/*Willdenowia*	MMYA-1/Myanmar, Inlay Lake	MT739758	[43]	MT739759, MT739761, MT739762, MT739763, MT739764, MT739767
			RB-CZ119A	MZ648322	this study	-
			RB-CZ119B	MZ648323	this study	-
			RB-CT1119-B	MZ648324	this study	-
	*Nitellopsis obtusa*		GEC4-1/Poland, Lake Lagowskie	MK791491	[59]	-
